# Circulating miRNA Signature as a Potential Biomarker for the Prediction of Analgesic Efficacy of Hydromorphone

**DOI:** 10.3390/ijms20071665

**Published:** 2019-04-03

**Authors:** Naoki Kiyosawa, Kenji Watanabe, Kaoru Toyama, Hitoshi Ishizuka

**Affiliations:** 1 Specialty Medicine Research Laboratories I, Daiichi Sankyo Co., Ltd., 1-2-58, Hiromachi, Shinagawa, Tokyo 140-8710, Japan; 2 Biomarker Department, Daiichi Sankyo Co., Ltd., 1-2-58, Hiromachi, Shinagawa, Tokyo 140-8710, Japan; watanabe.kenji.rv@daiichisankyo.co.jp; 3 Clinical Pharmacology Department, Daiichi Sankyo Co., Ltd., 1-2-58, Hiromachi, Shinagawa, Tokyo 140-8710, Japan; toyama.kaoru.fb@daiichisankyo.co.jp (K.T.); ishizuka.hitoshi.h8@daiichisankyo.co.jp (H.I.)

**Keywords:** miRNA, biomarker, opioid, cancer pain, hydromorphone

## Abstract

No practical biomarkers currently exist for the prediction of the analgesic efficacy of opioids. Previously, we reported circulating miRNA signatures differentially regulated by µ-opioid receptor (MOR) agonists in healthy subjects. We hypothesized that these miRNAs could be potential pharmacodynamic biomarkers to estimate MOR stimulation, and predict the efficacy of opioids; i.e., patients with low MOR stimulation may be more vulnerable to strengthening of the MOR signal upon hydromorphone treatment. To test this hypothesis, plasma samples were obtained from 25 patients with cancer pain prior to the initiation of hydromorphone treatment and the circulating miRNA levels were evaluated, focusing on four miRNAs (i.e., hsa-miR-423-3p, hsa-let-7a-5p, hsa-miR-26a-5p, and hsa-let-7f-5p) and four miRNAs (i.e., hsa-miR-144-3p, hsa-miR-451a, hsa-miR-215, and hsa-miR-363-3p) that were most clearly up and downregulated by hydromorphone and oxycodone. The patients were classified into two classes with putative high and low MOR signal, estimated based on the plasma miRNA signature. A significant correlation was observed between the analgesic efficacy and the putative MOR signal level, and patients with low MOR signal achieved better pain control (i.e., ΔVAS < 0) through hydromorphone. These results suggested that plasma miRNA signatures could serve as clinical biomarkers for the prediction of the analgesic efficacy of hydromorphone.

## 1. Introduction

Opioids have been widely used as analgesics to treat cancer pain [1]. However, considerable individual variability exists in response to opioids, in terms of both efficacy and safety [2]. Unfortunately, technical difficulties in the optimization of opioid prescription have created barriers that prevent patients from receiving adequate pain relief from opioids [3]. 

One of the factors affecting the response to opioid treatment is the development of tolerance associated with the long-term use of opioids [4]. The mechanism of tolerance development is attributed to pharmacogenetic factors, such as gene polymorphisms in drug metabolizing enzymes or the µ-opioid receptor (MOR) [5], or to the alteration of pharmacological dynamics due to repeated drug exposure. The latter mechanism often results in tolerance to other structurally similar opioids, termed cross-tolerance. Opioid switching aims to circumvent this cross-tolerance by changing to structurally distinct opioid drugs [6,7,8], but large individual variabilities exist in response to switched opioids. Thus, a precise medical strategy is desirable to maximize the benefits for individual patients [9], but there are currently no practical biomarkers to predict the analgesic efficacy of opioids. 

microRNAs (miRNAs) represent a class of non-coding small RNAs with an average length of 22 nucleotides [10]. miRNAs play crucial roles in various physiological and pathological processes [11], and their dysregulation is associated with disease conditions such as cancers of the liver, cardiovascular system, and central nervous system (CNS), or drug addiction [11,12,13]. In addition, a number of miRNAs associated with pain signaling are known to exist, and a number of pain-associated miRNAs have been reported in animal models and patients with fibromyalgia, migraine, and osteoarthritis [14,15,16,17,18,19]. Many miRNA studies in animal models of pain have been performed on neural tissues [15], although some have been performed on body fluids, including blood [20]. For example, extracellular let-7b and miR-599 were found to activate nociceptive sensory neurons to evoke pain via TLR7 and TRPA1 [21], which indicated that extracellular miRNAs can functionally modulate neuronal cells. 

Previously, we reported the circulating miRNA signatures in healthy subjects treated with hydromorphone or oxycodone [22]. Although the detailed molecular mechanisms have not yet been clarified, the differentially regulated miRNAs may reflect pharmacodynamic responses to MOR stimulation. Interestingly, some of the upregulated miRNAs in the plasma were reported to negatively regulate MOR functions (e.g., miR-146a-5p and miR-23b-3p), which suggested that the upregulation of these miRNAs may reflect negative feedback in response to MOR stimulation [22]. If this is the case, it may be possible to estimate MOR stimulation from the circulating miRNA signature, thereby enabling the prediction of analgesic efficacy of opioids prior to initiation of treatment (i.e., the greater the stimulation of the putative MOR signal, the lower potential for further MOR stimulation by opioid treatment). To test this hypothesis, we measured the plasma miRNA signatures in patients with cancer prior to the initiation of hydromorphone treatment who had switched from morphine drugs and evaluated whether the miRNA signature was associated with the analgesic efficacy outcome of hydromorphone treatment.

## 2. Results

### 2.1. Selection of MOR-Regulated miRNAs from Previous Healthy Subject Study Data

**Figure 1:**
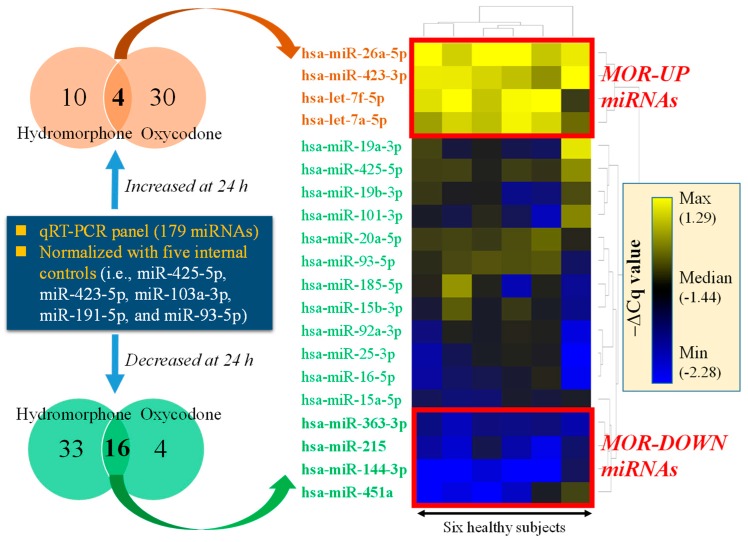
Selection of miRNAs as putative pharmacodynamic biomarkers reflecting µ-opioid receptor (MOR) stimulation. Circulating microRNA (miRNA) qPCR data were collected from healthy subjects treated either with hydromorphone or oxycodone [22]. The qPCR panel data were processed by using an internal control-based normalization method. Four and sixteen circulating miRNAs were identified as differentially regulated miRNAs commonly regulated by both hydromorphone and oxycodone. The heat map represents hierarchically clustered −ΔCq values measured in six healthy subjects treated with hydromorphone. Four “MOR-UP” (has-miR-423-3p, has-let-7a-5p, miR-26a-5p, and hsa-let-7f-5p) and four “MOR-DOWN” (has-miR-144-3p, has-miR-451a, has-miR-215, and has-miR-363-3p) miRNAs, which showed the clearest changes in expression, were selected and evaluated for their predictive potentials for the analgesic efficacy of hydromorphone in the study of patients with cancer.

The outline of the miRNA selection procedure is presented in Figure 1. The qPCR panel data comprised 179 miRNAs collected in healthy subject studies, and the data were processed with the internal control normalization method. The lists of differentially regulated miRNA (i.e., *q* < 0.05 between 24 h after treatment vs. before treatment with either hydromorphone or oxycodone) are provided in Tables S1 and S2. Four miRNAs, namely hsa-miR-423-3p, hsa-let-7a-5p, hsa-miR-26a-5p, and hsa-let-7f-5p, were commonly upregulated by both hydromorphone and oxycodone. Of these, hsa-miR-423-3p and hsa-let-7a-5p were also identified as commonly upregulated miRNAs using the global normalization method [22], and both hsa-miR-26a-5p and hsa-let-7f-5p were identified as upregulated miRNAs by hydromorphone using the global normalization method [22]. In contrast, 16 miRNAs were commonly downregulated by both hydromorphone and oxycodone. Of these, hsa-miR-144-3p, hsa-miR-451a, hsa-miR-215, and hsa-miR-363-3p exhibited the clearest downregulation by hydromorphone (Figure 1), and all these four miRNAs were also identified as commonly downregulated miRNAs by using the global normalization method [22]. 

In summary, we selected four upregulated miRNAs (has-miR-423-3p, has-let-7a-5p, miR-26a-5p, and hsa-let-7f-5p; termed MOR-UP miRNAs) and four downregulated miRNAs (has-miR-144-3p, has-miR-451a, has-miR-215, and has-miR-363-3p; termed MOR-DOWN miRNAs) as potential pharmacodynamic biomarkers that reflect MOR stimulation. All eight miRNAs were found to derive from bona fide miRNA genes, according to the MirGeneDB search results [23].

### 2.2. Study of Patients with Cancer

#### 2.2.1. Demographics of Patients with Cancer

**Figure 2:**
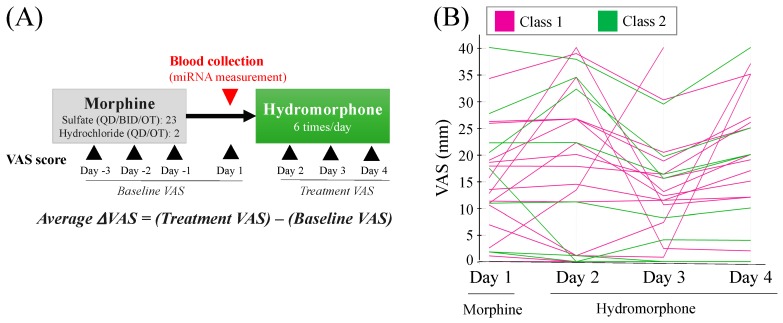
Study outline and pain intensities in patients with cancer. (**A**) Study outline. Cancer patients had received treatment with morphine for at least three consecutive days and were switched to hydromorphone treatment. The Visual Analog Scale (VAS) score was recorded once daily as a measure of subjective pain intensity. The Baseline VAS score was calculated as an average of the VAS scores from day −3 to day 1 (before the initiation of hydromorphone treatment). ΔVAS score, which represents the change in pain intensities after hydromorphone treatment, was calculated by the subtraction of the values of Baseline VAS from an average of VAS scores between day 2 and day 4 (Treatment VAS). Blood for miRNA analysis was collected from 25 patients before the initiation of hydromorphone treatment (day 1). (**B**) The time-course profiles of VAS scores for individual patients. The colors represent patient classes, which were determined from the plasma miRNA signature of the patients as presented in Figure 3A.

The outline of the study of patients with cancer [24] is presented in Figure 2A, with the demographics of the patients for the miRNA analysis presented in Table 1. There was no significant difference in age, baseline Visual Analog Scale (VAS) score (i.e., average of VAS scores from days −3 to 1), and rescue dose frequency (i.e., from days 1 to 4) between the two dose conversion groups: 21 patients received daily 60 mg/day morphine and four patients received 90 mg/day morphine treatments, who had achieved pain control with morphine and were randomly allocated 1:1 to hydromorphone immediate-release tablets at a conversion ratio of either 1:5 or 1:8. Severe adverse events (SAEs) were recorded for four patients during the study. 

#### 2.2.2. Analgesic Efficacy of Hydromorphone 

The time courses of the VAS scores for the 25 patients are presented in Figure 2B. No association was found between the dose conversion ratio (i.e., 1:5 or 1:8) and the analgesic efficacy potency of hydromorphone, which was consistent with the results for the full patient population reported previously [24]. For two patients, ΔVAS scores could not be calculated due to missing VAS scores during the study.

#### 2.2.3. Prediction of Analgesic Efficacy of Hydromorphone from Circulating miRNA Signatures 

**Figure 3:**
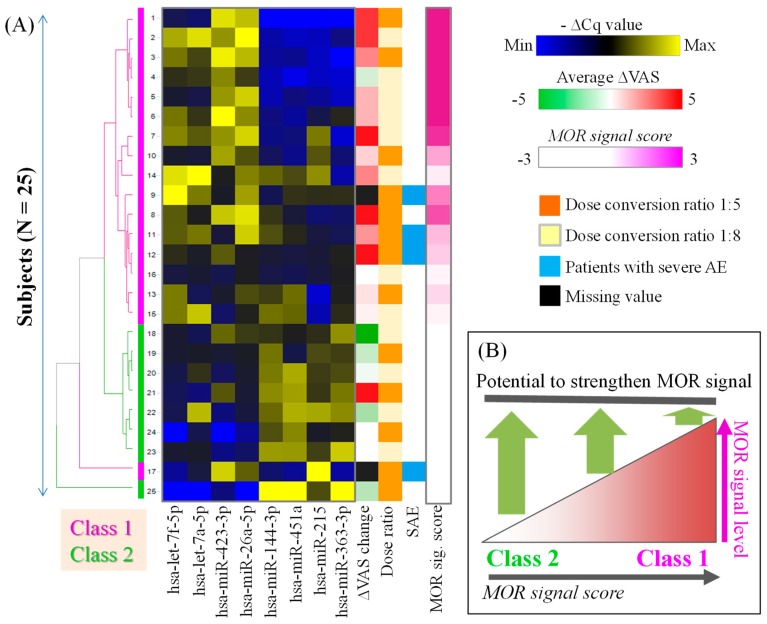
Circulating miRNA signatures of patients with cancer. (**A**) The miRNA signatures of patients with cancer before the initiation of hydromorphone treatment (i.e., day 1). The heat map of hierarchically clustered −ΔCq values for MOR-UP and MOR-DOWN miRNAs is presented; yellow and blue represent high and low blood miRNA levels, respectively. The MOR signal score is calculated from the subtraction of the sum of ΔCq for MOR-UP miRNAs from the ΔCq of the MOR-DOWN miRNAs. The patients with high and low MOR signal score values were classified into class 1 and class 2, respectively. The overall trend was that class 1 patients had higher ΔVAS scores than class 2 patients. (**B**) The putative model for the relationship between the MOR signal score and the potential for hydromorphone to exert analgesic efficacy.

The plasma miRNA levels prior to the initiation of hydromorphone treatment in the 25 patients with cancer are presented in Table 2 (note that plasma miRNA levels were higher when −ΔCq values were higher). The patients were classified into two classes by hierarchical clustering analysis of the plasma miRNA levels as presented in Figure 3A. Briefly, the class 1 patients, with high MOR signal scores, exhibited plasma miRNA signatures similar to those in healthy subjects treated with hydromorphone or oxycodone; and the class 2 patients, with low MOR signal scores, exhibited the opposite miRNA signatures to those of class 1 patients. 

A putative model for the relationship between the MOR signal score and the potential to exert further analgesic efficacy by hydromorphone treatment is shown in Figure 3B. In this model, class 1 patients, with high MOR signal score, may possess less potential for further stimulation of MOR, resulting in a poor response to hydromorphone. In contrast, class 2 patients with low MOR signal score may possess the potential for further stimulation of MOR, resulting in a good response to hydromorphone. 

**Figure 4:**
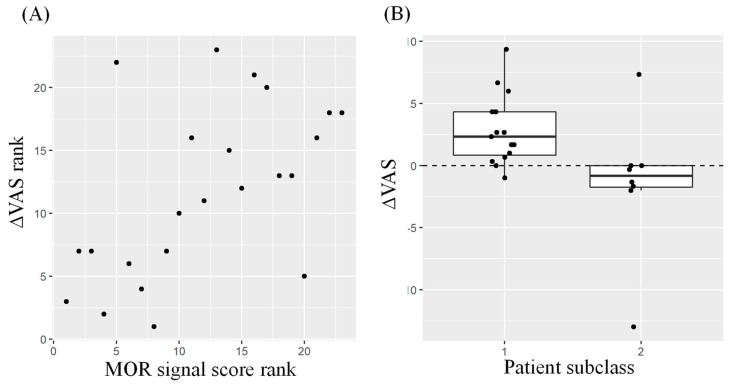
Analgesic efficacy and MOR signal score. (**A**) Rank order plots for ΔVAS scores and MOR signal scores. Significant rank order correlation was observed between ΔVAS and MOR signal score (*P* < 0.01 by Spearman’s correlation test). (**B**) Box plots for ΔVAS scores of class 1 patients (putative poor responders based on MOR signal score) and class 2 patients (putative good responders). Significant enrichment of patients with ΔVAS > 0 (class 1) and ΔVAS ≤ 0 (class 2) was indicated by Fisher’s exact test (*P* < 0.01).

As presented in Figure 4A, significant correlation was demonstrated between ΔVAS and MOR signal score (*P* < 0.01 by Spearman’s test). In addition, it was demonstrated that patients with ΔVAS > 0 were significantly enriched in class 1 (*P* < 0.01 by Fisher’s exact test) (Figure 4B). 

## 3. Discussion

In the present study, we tested our hypothesis that the plasma miRNA signature of patients with cancer was predictive of the analgesic efficacy of hydromorphone. We selected eight miRNAs in plasma that displayed the largest expression changes after both hydromorphone and oxycodone treatment in previously published healthy subject studies [22] (Figure 1). In the study of patients with cancer, those who had been treated with morphine were classified into two classes based on their plasma miRNA signatures prior to initiation of hydromorphone treatment (Figure 3A). The patients in class 1 exhibited similar miRNA signatures to those of healthy subjects treated with hydromorphone (i.e., high MOR signal score), which suggested that MOR was stimulated in those patients. We hypothesized that these class 1 patients may possess a lower potential to strengthen the MOR signal by additional hydromorphone treatment (Figure 3B). In contrast, patients in class 2 exhibited opposite miRNA signatures to those in class 1 (i.e., low MOR signal score), which indicated that the MOR signals in these patients were estimated to be low, and that there may be more potential to strengthen the MOR signal by hydromorphone treatment (Figure 3B). In support of this hypothesis, significant correlation was observed between the MOR signal scores and ΔVAS scores (Figure 4A). Furthermore, it was demonstrated that patients with ΔVAS > 0 (i.e., relatively poor responders) were significantly enriched in class 1 (Figure 4B). Collectively, these results indicated that the circulating miRNA signature appeared to reflect the MOR stimulation level, and that the MOR signal score indicated the capacity for further stimulatory potential of MOR prior to the initiation of hydromorphone treatment. 

The use of miRNAs as clinical biomarkers offers a number of advantageous characteristics [25]. For example, miRNAs are remarkably stable in body fluids such as blood [26,27]. However, the lack of reproducibility in miRNA signatures among different studies is of concern [12]. This may be partially attributable to the different platforms used for miRNA measurement [28]. To avoid this concern, we used the same qPCR panel platform in both this present study of patients with cancer and our previous studies of healthy subjects [22]. The selection of a normalization method for the qPCR data of circulating miRNAs is another confounding factor, as there is no currently established ‘gold standard’ normalization method [29]. Nonetheless, both the internal control-based and global normalization methods provided fairly consistent differentially regulated miRNAs from the qPCR data collected in healthy subject studies, which supported the notion that the selected eight miRNAs would provide robust results irrespective of a difference in normalization methods. To the best of our knowledge, this is the first report to present potential blood biomarkers with the ability for objective prediction of the analgesic efficacy of opioids. Our data suggested the promising potential of circulating miRNAs to realize the precision medicine of opioid therapy.

While our findings suggested the intriguing potential of circulating miRNAs as a biomarker to predict opioid efficacy, there were a number of limitations to this study. The first and the most serious concern was that the number of investigated patients was too small to draw robust conclusions. The range of pain intensities represented as VAS scores was quite narrow among patients; accordingly, the overall improvements in pain intensities (i.e., ΔVAS scores) were inevitably small. We invented a very simple model (i.e., MOR signal score) to estimate putative MOR stimulation in the present study; however, with the accumulation of relevant data from a larger number of patients exhibiting a wider range of pain intensities, the development of an optimized prediction model with rational cut-off values should be achieved. 

Second, the characteristics of the selected eight miRNAs should be clarified. The biological and pharmacological functions of the selected miRNAs are still largely unknown, although additional data from cutting-edge bioinformatics techniques should provide novel insights, such as target mRNAs and association with diseases [30,31]. In addition, the accumulation of biological knowledge for the miRNAs is required, such as potential circadian rhythm, confounding factors affecting variabilities in expression (e.g., food, comorbidity, age, gender, and cancer type), and the time-course of plasma levels following hydromorphone treatment. In addition, the tissue source of the circulating miRNA is unknown at present; these circulating miRNAs may be derived not only from CNS but from peripheral tissues and even blood cells where MOR is expressed [32,33]. The utilization of peripherally acting MOR agonists with weaker effects on the CNS [34] may partly answer these research questions. 

Third, a number of technical uncertainties need to be addressed to establish a robust and reliable clinical diagnostic system. For example, although residual platelets may confound miRNA signatures in the plasma samples [35], we did not perform a centrifugations procedure to remove platelets in our study. While the selected eight miRNAs appeared to successfully provide meaningful information in studies of both healthy subjects [22] and in the present study, further optimization and standardization of experimental procedures should be pursued. 

It is unknown if the predictability of the analgesic efficacy of hydromorphone by circulating miRNAs may also apply for other opioid drugs. In our previous study, a number of differentially regulated miRNAs overlapped between hydromorphone and oxycodone [22], but some differences between these drugs were still observed. If these differences reflected drug-specific mechanisms, contents of miRNA signature biomarkers may need to be customized for every opioid drug. Further data accumulation for various structurally distinct opioid drugs may address this question. 

In conclusion, we demonstrated the potential of circulating miRNA signatures as biomarkers to predict the analgesic efficacy of hydromorphone in patients with cancer. Further knowledge of both the biological and technical aspects of this process is required to realize the precision medicine of opioid therapies.

## 4. Materials and Methods 

### 4.1. Previously Published Studies in Healthy Subjects

#### Selection of miRNAs to Predict Analgesic Efficacy of Hydromorphone

Previously, we measured 179 circulating miRNAs in healthy subjects before and 24 h after a single treatment of either hydromorphone or oxycodone by using a Serum/Plasma Focus microRNA PCR Panel (Qiagen, Venlo, Netherlands), and processed the data by using a global mean normalization method [22]. In general, the global mean normalization method is effective when a sufficient number of target miRNAs are measured [36]; however, practically, the number of target miRNAs is usually small, and in such cases global normalization is not appropriate. Accordingly, in the present study, we re-analyzed the qPCR panel data by using an internal control-based normalization method. For the internal controls used for the normalization process, we selected five miRNAs (miR-425-5p, miR-423-5p, miR-103a-3p, miR-191-5p, and miR-93-5p) based on literature information, including the vendor’s publication [36,37,38]. The Cq values for the target miRNAs were subtracted from the average Cq values of the five internal controls to obtain the corresponding ΔCq values. The ΔCq values for all miRNAs obtained at pretreatment and at 24 h after the drug treatment were subjected to paired t-tests using Benjamini and Hochberg’s method to control the false discovery rate. 

From the differentially regulated miRNAs, we selected eight miRNAs (i.e., hsa-miR-423-3p, hsa-let-7a-5p, hsa-miR-26a-5p, hsa-let-7f-5p, hsa-miR-144-3p, hsa-miR-451a, hsa-miR-215, and hsa-miR-363-3p), as presented in Figure 1. Briefly, miRNAs commonly up or downregulated by both hydromorphone and oxycodone in the healthy subjects were determined. The change in expression of these miRNAs at 24 h after hydromorphone treatment in the healthy subjects was calculated by the subtraction of ΔCq at 0 h from ΔCq at 24 h after treatment. Four upregulated (“MOR-UP”) and four downregulated (“MOR-DOWN”) miRNAs were selected, as the expression changes in these miRNAs were more distinct than those in the rest of the miRNAs (Figure 1). For visualization purposes, qPCR data from healthy subjects treated with hydromorphone were subjected to hierarchical clustering analysis by using TIBCO Spotfire^®^ (Palo Alto, CA, USA) and presented as a heat map of the ΔCq values (Figure 1). 

### 4.2. Study in Patients with Cancer

#### 4.2.1. Study Outline

The outline of the study conducted on cancer patients is presented in Figure 2A. Plasma miRNA was measured in a double-blind, randomized comparative study (clinical trial registration ID: JapicCTI-132166), the primary objective of which was to confirm the morphine to the hydromorphone conversion ratio for hydromorphone immediate-release tablets in patients with cancer pain achieving adequate pain control with oral morphine [24]. The study was conducted as a multicenter, active-controlled, randomized, double-blind, parallel-group comparison study, enrolling 71 patients at 39 sites in Japan, in accordance with the ethical principles of the Declaration of Helsinki, the guidelines of Good Clinical Practice, and locally applicable laws and regulations. The study protocol, amendments, and informed consent forms were approved by the Institutional Review Boards of the respective study sites (list of the participating sites can be found in [24]). All subjects provided written informed consent prior to study commencement. miRNA measurements were conducted only for the patients who agreed to be tested. The exploratory miRNA research investigated 25 patients with cancer with an Eastern Cooperative Oncology Group (ECOG) performance status of ≤3. Patients who achieved pain control with morphine 60 mg/day and 90 mg/day for at least three consecutive days were randomly allocated 1:1 to hydromorphone immediate-release tablets (Daiichi Sankyo, Tokyo, Japan) at a dose-converted hydromorphone:morphine ratio of 1:5 or 1:8. Subjects in the conversion ratio 1:5 group received 12 mg/day of hydromorphone when their oral morphine dose was 60 mg/day during the pre-treatment observation period, and hydromorphone 18 mg/day when their oral morphine dose was 90 mg/day. Similarly, the subjects in the conversion ratio 1:8 group received hydromorphone 7.5 mg/day (oral morphine dose at 60 mg/day during the pre-treatment observation period) or 12 mg/day (oral morphine dose at 90 mg/day during the pre-treatment observation period). Hydromorphone was administered to patients six times daily. The minimum intervals from the last morphine treatment to the initiation of hydromorphone treatment were set as 20 h, 8 h, and 3 h for the morphine drugs prescribed at daily dose frequencies of once daily, twice daily, and six times daily, respectively. Rescue medication by morphine hydrochloride (1/6 of the dose level of that of medicated morphine; i.e., 10 mg morphine hydrochloride for patients medicated with 60 mg/day morphine) was allowed during the study period. The blood samples for miRNA measurement were collected before the initiation of hydromorphone treatment on day 1 (Figure 2A). 

#### 4.2.2. miRNA qPCR and Data Analysis

qPCR experiments were conducted at Takara Bio Inc. (Kusatsu, Shiga, Japan). Venous blood was collected into vacuum blood collection tubes containing EDTA-2K as a coagulant, and the plasma samples were prepared. Small RNAs were extracted from 200 µL of plasma by using the miRNeasy Mini Kit (Qiagen, Venlo, the Netherlands) in accordance with the manufacturer’s instructions. Single-stranded cDNA was synthesized by using Universal cDNA Synthesis Kit II (Qiagen). In total, 179 circulating miRNAs were measured by using Serum/Plasma Focus microRNA PCR Panel (Qiagen) and LightCycler^®^ 480 Instrument II (Roche Diagnostics, Basel, Switzerland). The Cq value, which represents the PCR cycle at which the designated threshold amplification level was reached, was determined for all target miRNAs by using GenEx software (Qiagen) in accordance with the manufacturer’s instructions [39]. The target miRNAs with amplification levels that did not reach the designated threshold after 40 cycles of amplification were considered absent and were excluded from further analysis. The normalization of qPCR panel data was conducted by using the internal control method with five miRNAs as internal controls (i.e., miR-425-5p, miR-423-5p, miR-103a-3p, miR-191-5p, and miR-93-5p); these were selected based on literature surveys, including the vendor’s publication [36,37,38]. The average Cq values for the five internal control miRNAs were subtracted from the Cq values of the target miRNAs to obtain the corresponding ΔCq values. Hierarchical clustering analysis for the ΔCq values was performed by using TIBCO Spotfire^®^ software to classify the patients into subclasses of putative high and low MOR signals. 

#### 4.2.3. Calculation of MOR Signal Score

The “MOR signal score” represents the putative MOR stimulation based on plasma levels of MOR-UP and MOR-DOWN miRNAs. Briefly, the MOR signal score increased when the plasma levels of MOR-UP were higher and those of MOR-DOWN were lower. The score was calculated from the following: 

*MOR signal score* = −∑(ΔCq of MOR − UP miRNAs) + ∑(ΔCq of MOR − DOWN miRNAs)

#### 4.2.4. Evaluation of Pain Intensity

For pain intensity, VAS score was measured every 24 h (±2 h) during the study to provide an average of pain intensity levels within the last 24 h. Baseline VAS score was determined as the average VAS scores for day −3, day −2, day −1, and day 1 (within 2 h of the first hydromorphone dose). As a measure of the analgesic efficacy of hydromorphone, the average ΔVAS score for each subject was calculated by the subtraction of the Baseline VAS score from average VAS scores for days 2, 3, and 4 (treatment VAS score) (Figure 2A). 

#### 4.2.5. Statistical Analysis

Fisher’s exact test was conducted using R (version 3.5.1) to evaluate the enrichment of patients with ΔVAS > 0 for the patient classes 1 and 2, determined based on the plasma miRNA signature (Figure 3A), with *P* < 0.01 considered to indicate statistical significance. The box plot was drawn by using R. The rank-order correlation between ΔVAS scores and MOR signal scores was evaluated by Spearman’s test using R, and *P* < 0.01 was considered to indicate statistical significance. 

## Figures and Tables

**Table 1:** Demographics of patients with cancer.

Parameter	Dose Conversion Ratio	
	1:5 (*N* = 12)	1:8 (*N* = 13)
**Age, years (mean ± SD)**	67.5 ± 11.9	69.2 ± 8.2
**Gender**		
Male	10	9
Female	2	4
**Underlying diseases (tumor types)**		
Urinary/ reproductive	1	2
Lung	3	6
Gastrointestinal	6	2
Head and neck	0	1
Breast	1	1
Others	1	1
**Morphine daily dose level (mg)**		
60	11	10
90	1	3
**Baseline VAS (mm)**		
Average ± SD	14.9 ± 11.9	13.3 ± 7.3
Maximum	36.7	25.3
Minimum	1.7	0
**Rescue dose frequency during the hydromorphone treatment period (times)**		
Average ± SD	3.2 ± 6.0	1.0 ± 1.8
Maximum	19	6
Minimum	0	0

**Table 2:** −ΔCq values for the selected miRNAs and MOR signal score.

ID	MOR−UP miRNAs				MOR−DOWN miRNAs				MOR Signal Score	Average ΔVAS	Dose Ratio	SAE
	hsa-let-7f-5p	hsa-let-7a-5p	hsa-miR-423-3p	hsa-miR-26a-5p	hsa-miR-144-3p	hsa-miR-451a	hsa-miR-215	hsa-miR-363-3p				
#1	−2.7	−0.3	−1.0	0.2	−2.8	5.0	−5.6	−3.5	3.0	4.3	1:5	
#2	−1.7	0.9	−1.3	0.4	−2.0	5.4	−5.0	−2.9	2.7	4.3	1:8	
#3	−2.0	0.3	−0.9	0.1	−1.9	5.7	−5.1	−3.5	2.2	2.7	1:5	
#4	−2.3	0.2	−1.3	−0.3	−2.1	5.2	−5.1	−3.3	1.6	−1.0	1:8	
#5	−2.5	−0.1	−1.3	0.2	−2.0	5.8	−4.7	−3.2	0.5	1.7	1:8	
#6	−2.0	0.2	−0.9	0.0	−1.6	5.7	−4.1	−2.4	−0.2	1.7	1:8	
#7	−1.9	0.4	−1.3	0.2	−1.6	5.9	−2.7	−3.3	−0.9	6.0	1:8	
#8	−2.1	0.1	−1.1	0.3	−0.6	6.3	−4.4	−2.8	−1.2	6.7	1:5	
#9	−1.3	0.5	−1.8	0.1	−1.3	6.6	−3.6	−2.4	−1.9	NA	1:8	+
#10	−2.5	0.0	−1.2	−0.2	−1.3	5.8	−3.1	−2.8	−2.4	1.0	1:5	
#11	−2.1	0.5	−1.9	0.2	−0.4	6.5	−4.1	−2.7	−2.7	2.3	1:5	+
#12	−2.3	0.1	−1.5	−0.4	−1.0	6.2	−4.0	−2.5	−2.9	9.4	1:5	+
#13	−1.9	−0.2	−1.8	−0.2	−0.5	7.0	−5.1	−2.5	−3.0	0.7	1:5	
#14	−1.5	1.0	−1.7	−0.1	−0.1	6.8	−2.4	−3.1	−3.3	2.7	1:8	
#15	−1.9	0.8	−2.2	−0.4	0.0	7.0	−4.9	−2.4	−3.4	0.3	1:8	
#16	−2.6	0.0	−2.1	−0.4	−1.2	6.1	−4.0	−2.5	−3.4	0.0	1:8	
#17	−3.1	0.0	−1.1	−0.2	−1.5	5.7	−0.9	−3.1	−4.7	NA	1:5	+
#18	−2.5	−0.1	−1.4	−0.3	−0.6	6.4	−3.6	−1.6	−4.9	−13.0	1:8	
#19	−2.5	0.1	−1.9	−0.5	0.0	6.2	−3.3	−2.2	−5.4	−1.3	1:5	
#20	−2.6	0.2	−2.2	−0.5	0.1	7.5	−3.4	−2.1	−7.2	−0.3	1:8	
#21	−2.7	−0.3	−1.5	−0.6	0.2	7.4	−3.5	−1.8	−7.6	7.3	1:5	
#22	−2.7	0.7	−2.4	−0.7	−0.1	7.6	−2.1	−1.6	−8.8	−2.0	1:8	
#23	−2.5	0.1	−2.5	−1.2	0.4	7.4	−3.4	−1.1	−9.4	0.0	1:8	
#24	−3.8	−0.1	−3.6	−1.7	−0.4	7.2	−3.9	−2.5	−9.7	0.0	1:5	
#25	−3.8	−1.2	−2.4	−3.2	1.5	8.2	−3.3	−0.6	−16.4	−1.7	1:5	

Negative ΔCq values for the selected eight miRNAs, average ΔVAS scores, and MOR signal scores are presented. MOR signal score is calculated by the subtraction of ∑(ΔCq of MOR-UP miRNAs) from ∑(ΔCq of MOR-DOWN miRNAs). The data are sorted by MOR signal score. NA, data not available. Note that plasma miRNA levels are higher when −ΔCq values are higher. SAE, severe adverse event.

## Supplementary Materials

Supplementary materials can be found at https://www.mdpi.com/1422-0067/20/7/1665/s1.

## Abbreviations

MOR	μ-opioid receptor
VAS	Visual Analog Scale
miRNA	microRNA
CNS	Central nervous system
ECOG	Eastern Cooperative Oncology Group
SAE	Severe adverse event
